# 

**Published:** 1854-01

**Authors:** 


### INDEX TO VOL. I.

| | PAGE |
|---|---|
| Air and Exercise, | 35 |
| Anointing with Oil, | 79 |
| American Manners, | 88 |
| American Climate, | 93 |
| Air Passages, | 105 |
| Air Indispensable to Health, | 114 |
| Asthma Described, | 111–119 |
| A Rapping Dream, | 127 |
| Annual Ailments, | 145 |
| Allen's Great Dental Achievement, | 219 |
| Aims of Life, | 271 |
| Apples, fried, | 296 |
| | |
| Brandy and Throat Disease, | 74 |
| Baths and Bathing, | 76 |
| Benefit of Action, | 79 |
| Boarding Life Mischievous, | 85 |
| Bronchitis—what is it, | 106 |
| Bodenhamer on Cholera, | 212 |
| Beer Drinking, | 272 |
| Brain and Thought, | 287 |
| | |
| Coffee and its Use, | 236, 231, 6 |
| Clerical Letter, | 57 |
| Clergymen's Sore Throat, | 75 |
| Clergymen's Case, | 106 |
| Comparative Health of Cities, | 106 |
| Consumption—what is it, | 108 |
| Convict's Autobiography, | 114 |
| Cutting Tonsils Dangerous, | 117 |
| Croup Described, | 119 |
| Courtesies of Life, | 121 |
| Children of the Rich, | 123 |
| Christianity and Medicine, | 136 |
| Coughing in Consumption, | 138 |
| Children Governed, | 161 |
| Cholera—what is it, | 169 |
| Cheerfulness, | 220 |
| Cold Water, vs. Health, | 226 |
| Common Sense, | 27 & 227 |
| Corn Bread and Constipation, | 280 |
| Consumption and Climate, | 290 |
| | |
| Dietetic Recipes, | 120 |
| Doubtful Witness, | 157 |
| Do we ever Forget, | 263 |
| Dyspepsia and Vinegar, | 272 |
| Doctors and Lawyers, | 279 |
| Drowning Sensations, | 282 |

| | PAGE |
|---|---|
| Death not always Painful, | 285 |
| | |
| Editor's Address, | 1 |
| Eating and Drinking, | 6 |
| Exertive Exercise, | 25 |
| Earth and Mind Culture, | 163 |
| Effects of Imagination, | 249 |
| Elbow Room, | 293 |
| | |
| Fatuity of Old Age, | 84 |
| Food we Eat, | 11 & 225 |
| Fruitful Neighborhood, | 248 |
| Fruit Healthful, | 292 |
| Family Jars, | 295 |
| | |
| Heart Disease, | 17 |
| Horseback Exercise, | 40 |
| Hominy and its Use, | 68 |
| How to Sit, | 87 |
| How to get up Early, | 88 |
| How we Grow, | 117 |
| How to be a Man, | 155 |
| Hardest Mode to Die, | 162 |
| Homeopathy, | 217 |
| How to Effect Reforms, | 220 |
| Half a Century in Bed, | 259 |
| Health, Wealth and Religion, | 273 |
| How to avoid Consumption, | 297 |
| | |
| Ice House Model, | 288 |
| Incurable Insanity, | 287 |
| Inhalation in Lung Diseases, | 297 |
| | |
| Kindness the best Punishment, | 141 |
| Kindness to Children, | 268 |
| | |
| Longevity, cases of, | 30 & 80 |
| Lung Measurement, | 49 |
| Lungs, | 73 |
| Lady's Bath, | 76 |
| Lament for Health Neglected, | 89 |
| Laryngitis—what is it, | 106 |
| Looseness of Cholera | 183 |
| Life's Great Object, | 276 |
| | |
| Milk and its Use, | 291 & 7 |
| Mistake of going South, | 95 |
| Merchant's Case, | 109 |
| Measures, | 120 |
| March to the Grave, | 168 |

| | PAGE |
|---|---|
| Marriage, disparity in age,........ | 258 |
| Marry, who to,.................. | 275 |
| Marriages Happy,............... | 296 |
| New Shoes made Easy,........... | 71 |
| Neglect of Physical Education,..... | 118 |
| Octogenarian Letter,............. | 102 |
| Overworking,.................... | 233 |
| Physician, the good,.............. | 1 |
| Physical Cultivation,............. | 86 |
| Pitching,....................... | 94 |
| Perpetual Asthma,.............. | 111 |
| Patent Medicines Murderous,...... | 119 |
| Preventives of Cholera,.......... | 181 |
| Potatoes Cooked,................ | 237 |
| Preservation of Cranberries, Fruits, | 237 |
| Pickles Healthful,............... | 238 |
| Preserved Eggs,................ | 244 |
| Rich Christian Men,............. | 60 |
| Rich Men's Children,............. | 123 |

| | PAGE |
|---|---|
| Rage and Ruin,................. | 133 |
| Recipes, 12, 43, 87, 103, 120, 155, 235– | 246 |
| Rainy Day, Recipe for,........... | 279 |
| Spirometrology,................. | 49 |
| Small Pox,..................... | 66 |
| Shaving the Beard,.............. | 97 |
| Sick Head Ache,................ | 109 |
| Suicide by Starvation,............ | 113 |
| Speak Gently,.................. | 246 |
| Travelling for Health,............ | 14 |
| Theological Seminary,........... | 82 |
| Tobacco, its Use and End,........ | 100 |
| Throat Ail,..................... | 106 |
| Teeth Cleaning,................ | 78, 219 |
| Tea,.......................... | 6, 232 |
| The True Christian Doctrine,...... | 278 |
| Ventilation,.................... | 31 |
| Visiting Cards Poisonous,......... | 155 |
| Vinegar and Dyspepsia,.......... | 272 |
| Who Murdered Downie,.......... | 253 |
| Weight of Various Foods,........ | 289 |

### NOTICES OF BOOKS, PERIODICALS, &c.

PROSPECTUS of the INDEPENDENT.—Volume Sixth, 1854.—This well-known and widely circulated Journal, conducted by Pastors of Congregational Churches in New York and vicinity, has completed its fifth year.

It is now enlarged; is published in a quarto form, and contains sixteen columns, or 50 per cent. more reading matter than ever before, being THE LARGEST RELIGIOUS PAPER IN THE WORLD.

In addition to the regular editorial corps, the Rev. G. B. Cheever, D.D., the Rev. Henry Ward Beecher, Mrs. H. B. Stowe, the Rev. C. L. Brace, and "Minnie Myrtle," are stated contributors, engaged to write weekly, and will be assisted by most able correspondents at home and abroad, who will do all in their power to make this Journal an interesting RELIGIOUS and FAMILY PAPER.

TERMS.—Notwithstanding the immense addition of at least $8,000 to the yearly expenses of the paper, the price will remain the same,

$2 PER ANNUM, BY MAIL, $2 50 BY CARRIERS, if paid strictly in advance, or $2 50 if not paid within three months. No new names entered without the money.

AGENTS.—Clergymen and Postmasters are authorized Agents, and are solicited to engage in the work of extending our circulation. Fifty Cents commission on each new subscriber will be allowed them.

Any person wishing to subscribe, will please enclose in an envelope TWO DOLLARS, and address JOSEPH H. LADD,

Publisher of The Independent, No. 10 Spruce st., New York,

Prepaying postage; and money so sent, will be considered at our risk.

The paper will be sent in exchange for one year to any newspaper or monthly periodical that will publish this prospectus, including this notice. New York, Jan. 5, 1854.

---

A PAPER FOR YOUR FAMILY.

### New Series—New Attractions—New Type.

### THE HOME JOURNAL FOR 1854.

In consequence of the great and continually increasing demand for this elegantly-printed, widely-circulated and universally popular Family Newspaper, we have, heretofore, been unable to furnish the back numbers to only a very limited extent. To avoid this disappointment in future, we shall, on the first of January next, print such an increased edition as will enable us to supply new subscribers from that date. Besides the original productions of the Editors—the Foreign and Domestic Correspondence of a large list of contributors—the spice of the European Magazines—the selections of the most interesting publications of the day—the brief novels—the piquant stories—the sparkling wit and amusing anecdote—the news and gossip of the Parisian papers—the personal sketches of public characters—the stirring scenes of the world we live in—the chronicle of the news for ladies—the fashions and fashionable gossip—the facts and outlines of news—the pick of English information—the wit, humor and pathos of the times—the essays on life, literature, society and morals, and the usual variety of careful choosings from the wilderness of English periodical literature, criticism, poetry, etc.—several new and attractive features of remarkable interest will enrich and give value to the new series of the work.

TERMS—For one copy, $2; for three copies, $5, or one copy for three years, $5—always in advance.

Subscribe without delay. Address MORRIS & WILLIS.

---

### "USES AND ABUSES OF AIR,"

Showing its influence in sustaining life and producing disease, with remarks on the ventilation of houses; 12 mo., 249 pages, with numerous plates, plans of houses,

&c.; its concluding sentence is, "*A pure atmosphere, the first requisite for healthy bodies and sound minds.*"

I consider Dr. Griscom's book as the most useful publication of the kind published within the last ten years. No intelligent man can rise from its perusal without having received valuable hints and impressions which will last for life, and which, if practically improved, would add to his age and healthfulness. No clergyman, no lawyer, no large family of growing children, should be without it or its teachings. Price Seventy-five cents.

---

"HUFELAND'S ART OF PROLONGING LIFE" has just been republished by Ticknor, Reed & Fields, Boston; 328 pages, 12 mo.

The enterprising publishers have done a public service in the reissue of this useful book. It is thus noticed by the *American Medical Monthly* of New York, of which Edward H. Parker, M.D., is Editor, assisted by the Faculty of the New York Medical College, 80 pages, 8 vo., sent free of postage for three dollars a year in advance.

"It treats of the means that shorten and those which lengthen life. The work is timely. Our youth are crowding on a great amount of steam, and traveling through the periods of individual life at prodigious speed. A perusal of Hufeland's judicious maxims will tend to make us take matters more coolly, to eat more slowly, sleep better and longer, work to better advantage, by learning to think before acting, lay together less kindling wood for future repentance, and live to a better old age."

This well-written notice indicates the point to which the attention of educated medical men is directed, the preservation of the health and constitutions of the young, showing that the true physician is not only ambitious of curing those who are sick, but that he disinterestedly strives for the prevention of disease; and from the endorsement of such names as Green and Carnochan, the public have a guarantee that the *American Medical Monthly* will in this, as in others, labor for what "pertains to the welfare of our public institutions, and the advancement of professional excellence and knowledge. Its design is not to supplant existing Journals, but to cultivate a field which it is believed has hitherto been neglected."

### NOTICES.

Prepayment in all cases is indispensable. The receipt of the "Journal" will be presumptive evidence that it is paid for, or sent as a specimen, intimating, in the latter case, that it is the special desire of the Editor that the person to whom it is sent should become a subscriber without delay.

The postage of the Journal is only six cents a year, if pre-paid at the Post Office of each subscriber.

---

### TO CORRESPONDENTS.

All orders or communications, books for notice or review, correspondence, &c., must be addressed simply to

"Hall's Journal of Health, New York,"

or, if within the city, to the office of the Editor, 37 Irving Place, New York, near Union Square, until May 1st next, and thereafter, at his private residence, 42 Irving Place.

A single specimen number will be sent by the Editor, post-paid, to his personal friends and former patients in the different states of the Union; and he desires it to be considered as his particular desire, that such would become subscribers: thus affording him, from time to time, the satisfaction of knowing that they "STILL LIVE," and that his labors are tending to promote the health and consequent happiness of those whom he has once pleasurably known.

One specimen number will be also sent to a number of clergymen, for it originated in a desire to promote their good, and the Editor will endeavor to conduct it in such a manner, that any pains they take to procure subscribers, especially from among the young men and young women of their congregations, will be compensated in the permanent healthfulness of those who are to take their places when their own labor is done, and they have gone up to their reward.

The Journal will be sent also to some of the public men in the country, whose health and length of life are believed to be necessary to the highest interests of the communities in which they reside. The postage on all specimen numbers will be prepaid at the New York Post Office, and it is specially requested, to preserve the Editor from loss, that the specimen number be returned, when not taken, to address of "Hall's Journal of Health, New York."

A PREMIUM of twenty-five dollars will be paid to any subscriber who will write, from his own experience, the best description of How I LOST MY HEALTH, to contain within fifty, of eight hundred words, on or before the first day of June next. A sealed envelope should accompany the manuscript, containing the full name and address of the author, none of which will be broken, except that of the successful one; these envelopes will be returned by mail, unopened and prepaid, to each one who desires it. The manuscripts will be retained, unless called for.

---

### CONTENTS.

☞ The "Journal" will be sent one year to any established newspaper or periodical which will notice its appearance, and copy the contents of the January number—*provided* such publication is sent, with the article scored, to address of

"HALL'S JOURNAL OF HEALTH, NEW YORK."

